# Molecular mechanisms of action and potential biomarkers of growth inhibition of dasatinib (BMS-354825) on hepatocellular carcinoma cells

**DOI:** 10.1186/1471-2407-13-267

**Published:** 2013-05-30

**Authors:** Alex Y Chang, Miao Wang

**Affiliations:** 1 Johns Hopkins University, Baltimore, USA; 2 Johns Hopkins Singapore International Medical Centre, 11 Jalan Tan Tock Seng, Singapore 308433, Singapore

**Keywords:** Src kinase, Mechanism of inhibition, Dasatinib, Biomarker, Hepatocellular carcinoma

## Abstract

**Background:**

Molecular targeted therapy has emerged as a promising treatment of Hepatocellular carcinoma (HCC). One potential target is the Src family Kinase (SFK). C-Src, a non-receptor tyrosine kinase is a critical link of multiple signal pathways that regulate proliferation, invasion, survival, metastasis, and angiogenesis. In this study, we evaluated the effects of a novel SFK inhibitor, dasatinib (BMS-354825), on SFK/FAK/p130CAS, PI3K/PTEN/Akt/mTOR, Ras/Raf/MAPK and Stats pathways in 9 HCC cell lines.

**Methods:**

Growth inhibition was assessed by MTS assay. EGFR, Src and downstream proteins FAK, Akt, MAPK42/44, Stat3 expressions were measured by western blot. Cell adhesion, migration and invasion were performed with and without dasatinib treatment.

**Results:**

The IC_50_ of 9 cell lines ranged from 0.7 μM ~ 14.2 μM. In general the growth inhibition by dasatinib was related to total Src (t-Src) and the ratio of activated Src (p-Src) to t-Src. There was good correlation of the sensitivity to dasatinib and the inhibition level of p-Src, p-FAK576/577 and p-Akt. No inhibition was found on Stat3 and MAPK42/44 in all cell lines. The inhibition of cell adhesion, migration and invasion were correlated with p-FAK inhibition.

**Conclusion:**

Dasatinib inhibits the proliferation, adhesion, migration and invasion of HCC cells in vitro via inhibiting of Src tyrosine kinase and affecting SFK/FAK and PI3K/PTEN/Akt, but not Ras/Raf/MEK/ERK and JAK/Stat pathways. T-Src and p-Src/t-Src may be useful biomarkers to select HCC patients for dasatinib treatment.

## Background

Hepatocellular carcinoma (HCC) is one of the most common malignancies worldwide accounting for 500,000 ~ 600,000 deaths per year [1]. The major obstacles in the treatment of HCC are low resectable and high recurrence rates in patients with early disease and a poor response to chemotherapy and radiation in advanced stage disease [2,3]. In addition, a majority of HCC patients also have liver cirrhosis with poor liver functions and performance status, thus limiting their ability to receive treatment. In fact, the existing conventional chemotherapeutics are non-selective cytotoxic drugs with systemic side effects and no proven survival benefit. Therefore, there is often no effective therapy that can be offered to these patients [1,4]. In some series, up to 50% of patients with newly diagnosed HCC were only given supportive or palliative therapy. There is an urgent need to develop novel treatments for advanced HCC.

Targeted therapies that specifically inhibit pivotal molecular abnormalities have emerged as a promising approach for various cancers, including HCC [5]. Sorafenib, a dual inhibitor of Raf Kinase and VEGFR, is the only approved agent for treating advanced HCC. Sorafenib when compared to placebo prolongs the survival modestly by 2 to 3 months. Therefore, more efforts are necessary in the identification of new molecular targets to improve treatment further. One potential target is found in the Src family Kinase (SFK). C-Src, a non-receptor tyrosine kinase, has been found to be a critical component of multiple signaling pathways that regulate proliferation, invasion, survival, metastasis, and angiogenesis [6,7]. To carry out these activities, C-Src interacts with numerous cellular factors, including integrins, growth factor receptors, G-protein coupled receptors and cytokine receptors to initiate their downstream signaling cascades [8]. C-Src can cooperate with receptor kinases to signal through downstream molecules, such as PI3K/PTEN/Akt, Ras/Raf/Mek1/2/Erk1/2 and Stats [9-11]. C-Src also interacts with focal adhesion kinase (FAK), which plays an important role in integrin signaling and is highly expressed in many tumor cells, including HCC [12]. Tyrosyl phosphorylation of FAK interacts with multiple cellular proteins to modulate cell adhesion, migration and invasion [11].

Dasatinab (BMS-354825), a potent oral tyrosine Kinase inhibitor against the Src family Kinases, BCR-ABL, platelet derived growth factor receptor and c-Kit has demonstrated multiple effects on solid tumors and has been approved for use in patients with chronic myelogenous leukemia refractory or intolerant to imatinib [13] and in patients with Philadelphia chromosome-positive acute lymphoblastic leukemia [14]. Although there are active research studies evaluating the molecular mechanisms of dasatinib on human solid tumor cells such as prostate cancer, head and neck squamous cell carcinoma, non-small cell lung cancer, breast cancer, but the true regulatory mechanisms are still not fully understood, especially in HCC [15-21].

In this study, we hypothesize that dasatinib inhibits HCC by modulating SFK/FAK/p130CAS, PI3K//PTEN/Akt/mTOR, Ras/Raf/MAPK and/or Stats signaling pathways. The current investigation was undertaken to test this hypothesis.

## Methods

### Cell lines and cell culture

Human hepatocellular carcinoma (HCC) cell lines, HepG2, sk-Hep1, Hep3B were obtained from ATCC, HLE, HLF, Huh-7, HT-17, PLC/PRF/6 and Li-7 were provided by Institute of Molecular and Cell Biology of Singapore. All cell lines were cultured in Dulbecco’s Modified Eagle Medium [high Glucose (4.5 g/L), with Sodium Pyruvate and L-glutamin] (PAA Laboratories Cell Culture Products, Austria), containing 10% fatal bovine serum (FBS) (Invitrogen, USA), 1% antibiotic with 100 IU/ml Penicillin and 100ug/ml Streptomycin (Invitrogen, USA). Incubation condition was set at 37°C in a humidified atmosphere of 95% air and 5% CO_2_. The culture medium was changed 2 to 3 times a week and cells were passaged using trypsin/EDTA (Invitrogen, USA).

### Antibodies and reagents

Src rabbit monoclonal antibodies, β-actin, rabbit monoclonal antibodies against the phosphor-Src(Tyr416), phosphor-Akt(Ser473),  phosphor-MAK42/44(Thr202/Tyr204), phosphor-Stat3(Tyr705), phosphor-FAK576/577 were from Cell Signaling Technologies, Canada. Polyclonal antibody to phosphor-FAK861 was purchased from Invitrogen Corporation, Canada. Polyclonal goat anti-rabbit immunoglobulins/HRP was from Dakocytomation, Denmark. Recombinant human epidermal growth factor was purchased from Invitrogen Corporation, USA. Dasatinib was obtained from Bristol-Myers Squibb, Princeton, USA.

### Growth inhibition assay

Dasatinib was diluted in pure DMSO to obtain a stock solution of 10 mmol/L and stored in a −80°C freezer in aliquots. CellTiter 96 Aqueous Non-Radioaction cell proliferation Assay Kit (Promega Corporation, USA) was used for growth inhibition assays. 4000–10,000 HCC cells from 9 cell lines were plated in 96-well flat-bottomed plates and cultured for 24 hours (h). Cells were exposed to serially diluted dasatinib in DMEM with 1%FBS, for an additional 72 hours. 20 μl MTS/PMS solution was added into each well containing 100 μl of the culture medium. Then, the cells were incubated for 3 h at 37°C before measurement of absorbance at 490 nm with a Benchmark Plus microplate spectrophotometer (Bio-RAD, USA). Absorbance values were expressed as a percentage of that for untreated cells, and the concentration of dasatinib resulting in 50% growth inhibition (IC_50_) was calculated for each cell line. As reported by us previously, we arbitrarily defined the sensitive cell lines as having their IC_50_ ≤ 1uM and the resistant cell lines IC_50_ ≥1uM [22].

### EGF stimulation and dasatinib treatment

Briefly, approximately 2 × 10^5^ cells were seeded into 6-well plates in serum containing medium. After 24 h culture, cells undertook serum starvation for additional 24 h and then were exposed to 10 ng/ml EGF (Millipore, USA) for PLC/PRF/6 cells and 200 ng/ml for sk-hep1 cells for 5 min, 10 min, 15 min, 30 min, 1 hour. Finally the cells were harvested for western blotting analysis.

For dasatinib inhibition study, serum-starved cells were treated with various concentrations of dasatinib for 24 h prior to the addition of 20% FBS stimulation, and then were collected for western blotting analysis. In order to show that this treatment would not affect cellular viability, we selected sk-Hep1 and Huh-7 as the representative examples of the sensitive and resistant cell lines to dasatinib for the following experiment: 8000 cells were seeded into 96-well plate overnight, and then divided into 3 groups A, B and C before dasatinib treatment. Group A was serum-starved for 24 h, group B and C were incubated in culture medium with 1% FBS and 10% FBS respectively. After another 24 h dasatinib treatment MTS assay was used to determine the cell viability.

### Protein extraction and Western blotting

The cells were lysed for protein extraction using M-PER mammalian protein extraction reagent with protease inhibitor and phosphatase inhibitor (Thermo scientific, Pierce Biotechnology, USA). The total protein concentration was measured by BCA kit (Pierce Biotechnology, USA). Isolated proteins (35 μg/lane) were separated by 8% SDS-PAGE and transferred to a nitrocellulose membrane by the iblot device (Invitrogen Corporation, CA). The membranes were blocked with 5% BSA at room temperature for 1 h and then subjected to immunoblots using primary antibodies at 4°C overnight, followed by incubation with secondary goat anti-rabbit IgG conjugated to horseradish peroxidase for 1 h at room temperature. Labeled protein was visualized by chemiluminescence (Immobilon, Millipore Corperation, USA) and exposure x-ray film (Kodak, USA), using β-actin expression as the internal standard.

### Cell adhesion, migration and invasion assay

Cells were pretreated with dasatinib (1 μM) for 24 h after being starved overnight at 37°C in a humidified incubator containing 5% CO_2_. Cell adhesion assay was performed using the cell adhesion assay kit (Chemicon International, USA) by following the manufacturer instructions. Briefly, 96-well plates were coated with different Extracellular Matrix (ECM) proteins. Pretreated cells were re-suspended in assay buffer (Kit components) and seeded (1.5x10^5^) in each well. Plates were then incubated for 2 h at 37°C with 5% CO_2_. After removing the non-adherent cells and washing by assay buffer, cells were fixed and stained for 5 minutes, after washing 3–5 times with deionized water, the cell-bonded stain was solubilized and quantified with an ELASA plate reader (Benchmark Plus microplate spectrophotometer, Bio-RAD, USA), at 560 nm.

Cell migration assays was done by using the cell migration assay kit (Chemicon International, USA). Briefly, inserts with an 8 μm pore size polycarbonate membrane were utilized. 1.5 × 10^5^ cells were pretreated with dasatinib for 24 h and then seeded after washing off dasatinib into the inserts. Same number of untreated cells was used as control. All the inserts were put in the 24-well plate which was considered as the lower chamber, then DMEM with 10% FBS as the chemo- attractant was supplied in each wells. The cells were allowed to incubate at 37°C with 5% CO_2_ for 6 h and 16 h respectively. After that, cells in the inner surface of the inserts were gently removed. Cells that had migrated through the polycarbonate membrane were incubated with cell stain solution (kit components), then subsequently extracted and detected on a standard microplate reader (Benchmark Plus microplate spectrophotometer, Bio-RAD, USA), at 560 nm.

Cell invasion assay was processed by using the cell invasion assay kit (Chemicon International, USA). A 24-well tissue culture plate with cell culture inserts which contained an 8 μm pore size polycarbonate membrane was used. 1.5 × 10^5^ testing cells in serum free DMEM were plated into ECM coated insert, then DMEM with 10% FBS was placed in the 24-well plate as chemo attractants. After 48 h incubation, the cells were removed from the inner surface of the insert using a cotton-tipped swab. The cells that invaded through the ECM layer and clung to the bottom of the polycarbonate membrane were fixed and stained. The number of migrating cells per insert was captured microscopically.

### Statistical analysis

All the experiments were repeated at least 3 times. Data are reported as means ± SD. Correlation coefficient (r) was calculated by the Pearson product–moment correlation coefficient, and statistical significance (p-value) was analyzed using t approximation. The expression level of protein measured by western blot was analyzed by ImagJ software, p-values were calculated using the Students *t*-test.

## Results

### Growth inhibition by dasatinib in 9 HCC cell lines

**Figure 1:**
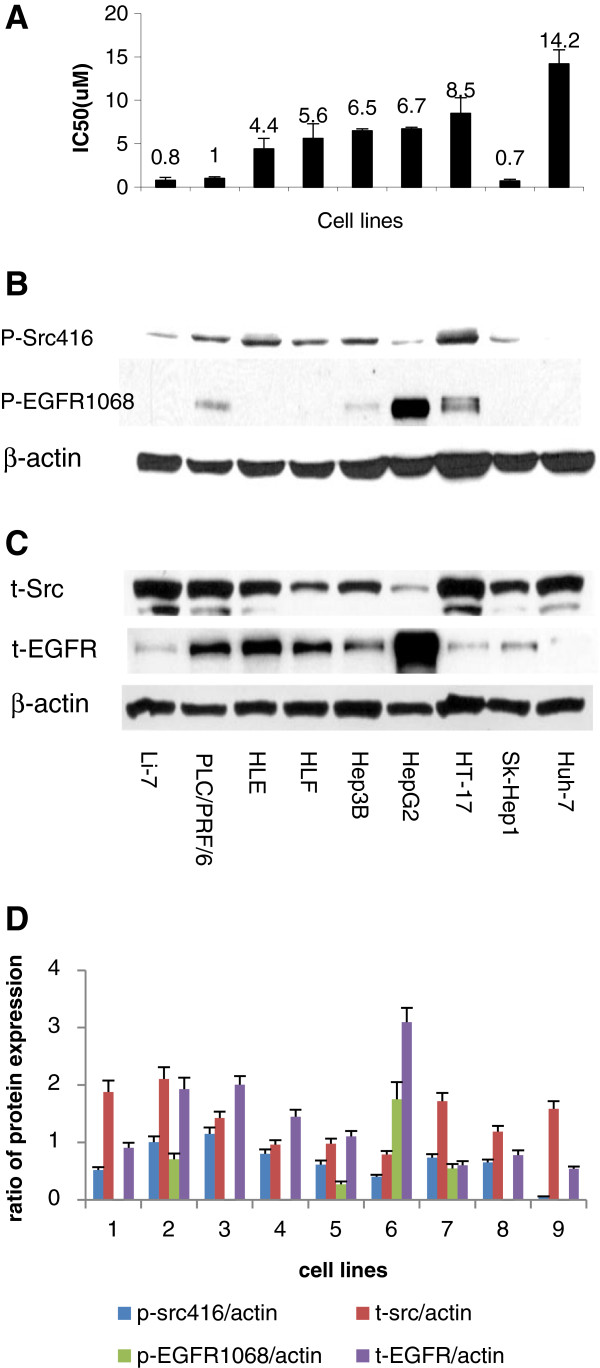
**Baseline protein expression as well as IC**_**50 **_**of dasatinib in HCC cell lines. A**, 9 HCC cell lines were exposed to the dedicated concentrations of dasatinib for 72 hours, and IC_50_ was tested by MTS. Results represent the mean (± SD) of three experiments. **B** and **C**, cell lysates were prepared from untreated HCC cell lines and subjected to western blot analysis with antibodies to p-Src416, t-Src, p-EGFR1068, t-EGFR and β-actin. **D**, the expression ratio of p-Src416, P-EGFR1068, Src and EGFR to β-actin was quantified by ImageJ software respectively. Results represented the mean (±SD) of three experiments.

The growth inhibition of each cell line was quantified by IC_50_ of dasatinib which ranged from 0.7 μM ~ 14.2 μM. Dasatinib showed a dose-dependent inhibition in all 9 HCC cell lines, Sk-Sep 1, Li-7, and PLC/PRF/6 were most sensitive with IC_50_ at or below 1 μM of dasatinib, while Huh-7 was most resistant (Figure 1A).

### Dasatinib inhibits Src activity and downstream signaling

**Figure 2:**
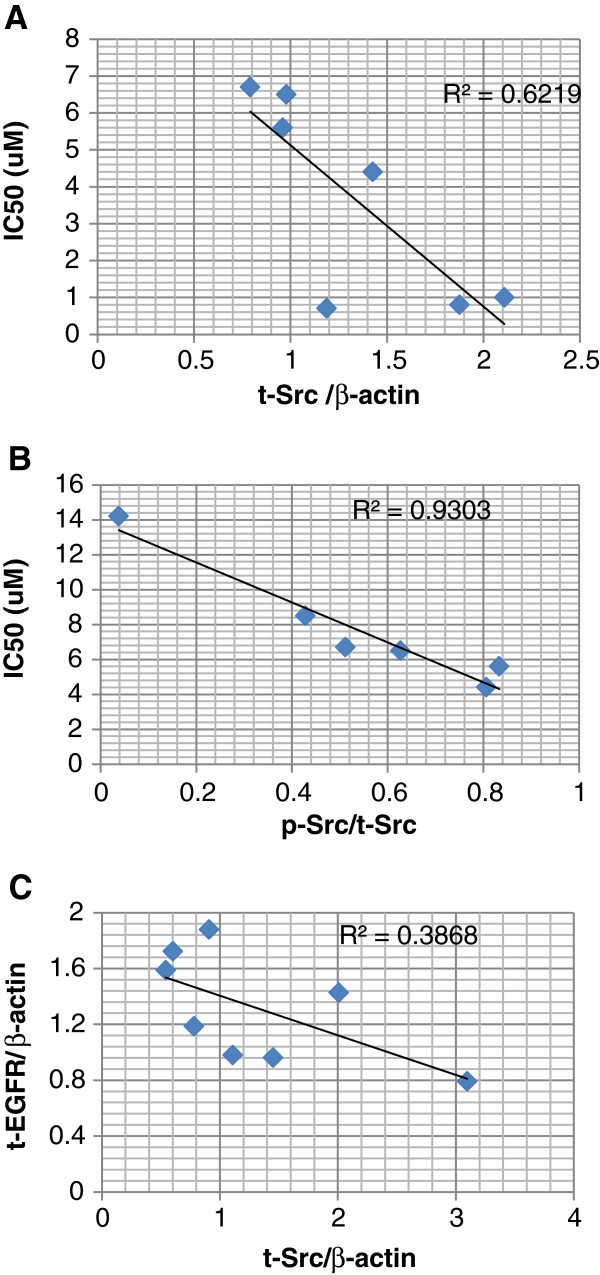
**Correlation between the growth inhibition by dasatinib and baseline protein expression. A**. The correlation between the growth inhibition by dasatinib and t-Src expression in HCC cell lines. 7 out of 9 studied cell lines showed significant correlation (r = −0.801, p = 0.03). **B**. The correlation between the IC_50_ of dasatinib and the ratio of p-Src/t-Src in 6 dasatinib resistant HCC cell lines. (r = −0.96, p = 0.001). **C**. The correlation between the expression level of Src and EGFR in 8 out of 9 HCC cell lines(r = −0.62, p = 0.05). All the studied protein expression were measured by western blot and analyzed by ImageJ.

**Figure 3:**
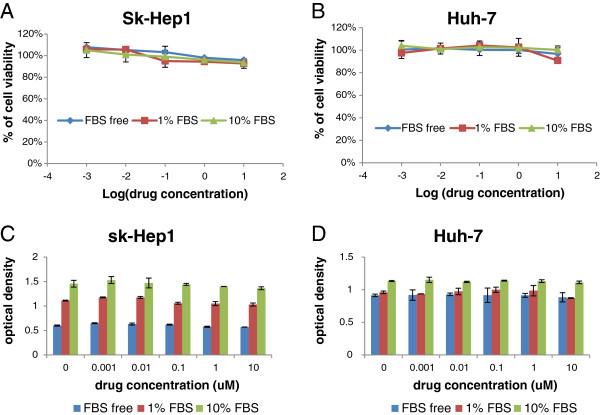
**The comparison of cell viability with different treatment condition.** Sk-Hep1 and Huh-7 cells were treated under three different conditions as described in methods. Results represented the mean (±SD) of three experiments. There was no influence of cell survival of sk-Hep1 (**A**) and Huh-7 (**B**) cells by dasatinib. Serum affected the cell concentration of sk-Hep1 (**C**) and Huh-7 (**D**) cells without the influence by dasatinib concentration.

The baseline levels of Src and activated Src (pY416-Src) were measured in 9 HCC cell lines by western blotting (Figure 1B and 1C). Except HT-17 and Huh-7 the rest of the cell lines showed significant correlation between growth inhibition by dasatinib (IC_50_) and the expression level of total Src (t-Src) (p < 0.05, Figure 2A). The higher the expression of t-Src, the more sensitive the HCC cell lines were to dasatinib. The average expression percent of p-Src in t-Src (p-Src/t-Src) for sensitive cell lines was significantly lower than that of resistant cell lines except for Huh-7 and HT-17 (p < 0.05). There was an extremely low expression of p-Src at base line in Huh-7 cells. In the 6 resistant cell lines we demonstrated that the specific activity of Src (the ratio of p-Src/t-Src) was significantly associated with the IC_50_ value of dasatinib. The lower the ratio of activity of Src (p-Src/t-Src), the more resistant the HCC cell lines to dasatinib (p = 0.001, Figure 2B). In 8 HCC cell lines the high levels of Src expression were significantly associated with low levels of EGFR expression (p = 0.05, Figure 2C). PLC/PRF/6 was the only cell line that expressed both high levels of t-Src and t-EGFR. The expression level of phosphorylated EGFR (p-EGFR) was only detected in 4 cell lines (PLC/PRF/6, Hep3B, HepG2 and HT-17).HT-17 showed the highest specific activity of EGFR (p-EGFR/t-EGFR) (Figure 1B and 1C). Figure 1D showed the quantity of t-Src, p-Src, t-EGFR and p-EGFR analyzed by software of ImageJ (Figure 1D). The cell viability of group A, B and C did not show any significant difference by various concentration of dasatinib in sk-Hep1 and Huh-7 cells (Figure 3A and 3B, p > 0.05). Although we showed serum affected the cell proliferation (Figure 3C and 3D, p < 0.05), it couldn’t affect the response of HCC cells to dasatinib.

**Figure 4:**
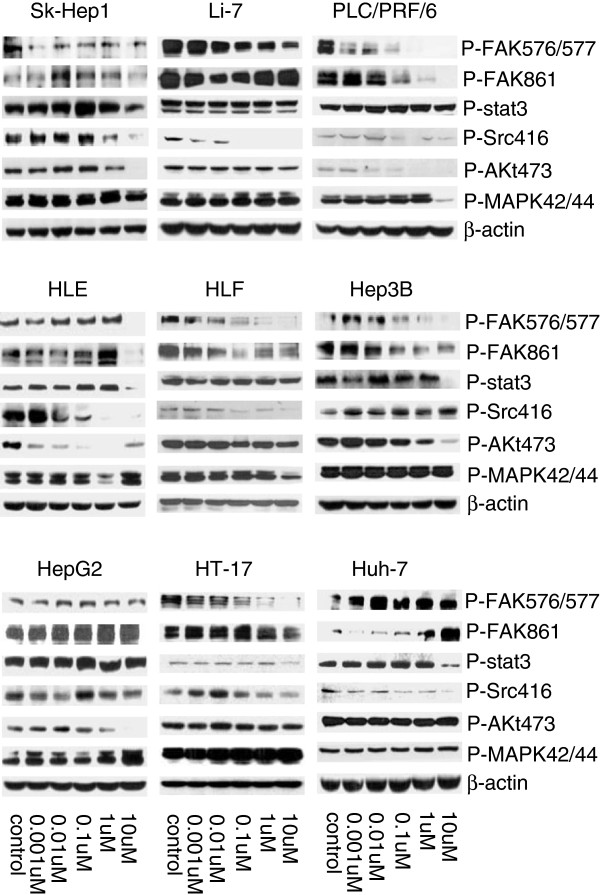
**The effect of dasatinib in cell signalling.** Western blot analysis with phosphorylated Src, FAK, Stat3, Akt and MAPK in HCC cell lines after 24 hours treatment of dasatinib. The cell lines were arranged according to their IC_50_ to dasatinib. The top three were most sensitive, the bottom 3 were least sensitive.

**Figure 5:**
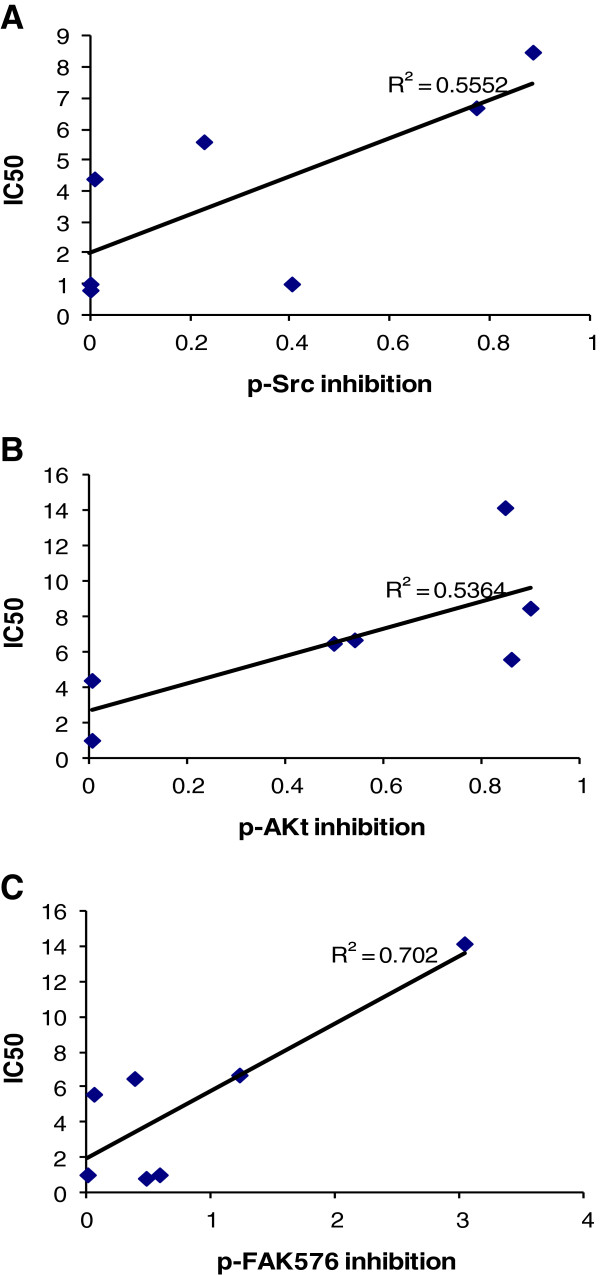
**Correlation between the IC**_**50 **_**of dasatinib and the inhibition on the activity of Src, Akt, FAK.** The inhibition levels of p-Src, p-Akt and p-FAK were measured when cells were treated with dasatinib at the dosage of 1uM and analyzed by ImageJ software. To analyze the correlation amongst the inhibition of different activated proteins by dasatinib, the inhibition of activated protein is calculated by the following formula, $\mathrm{for}\;\text{example}:\frac{\mathrm{pSrc}\left(D\right)/\beta -\text{actin}\left(D\right)}{\mathrm{pSrc}\left(C\right)/\beta -\text{actin}\left(C\right)}$, D for dasatinib treatment, C for control. **A**, 7 out of 9 studied HCC cell lines showed good correlation between IC_50_ and p-Src416 inhibition (r = 0.745, p = 0.03). **B**, 7 out of 9 studied HCC cell lines showed good correlation between IC_50_ and p-Akt473 inhibition (r = 0.732, p = 0.03). **C**, 7 out of 9 studied HCC cell lines showed good correlation between IC_50_ and P-FAK576/577 inhibition (r = 0.838, p = 0.01).

**Figure 6:**
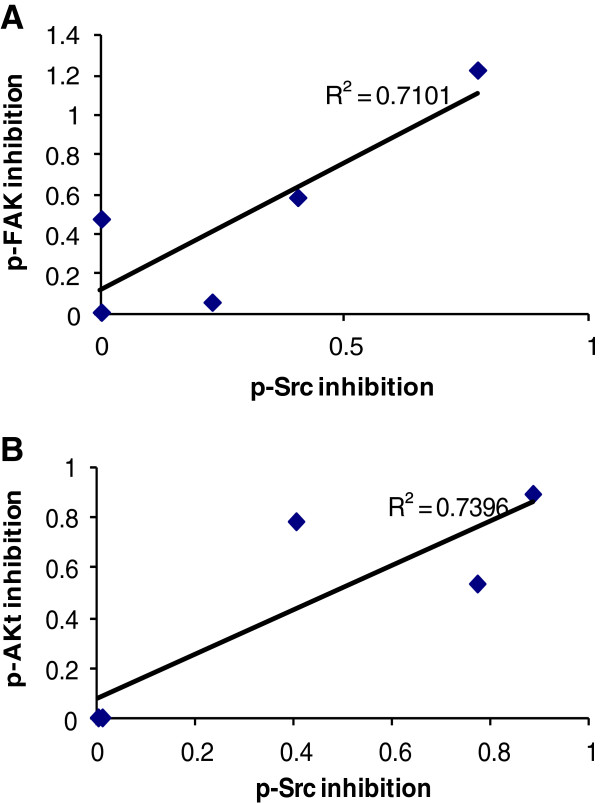
**Correlation of the expression level between p-FAK, p-Akt and p-Src inhibition after dasatinib treatment.** The methods of measurement and calculation is the same as Figure 5. **A**. 5 out of 9 HCC cell lines showed a good correlation between Src and FAK inhibition by dasatinib (r = 0.843, p = 0.04). **B**. 5 out of 9 HCC cell lines showed a good correlation between p-Src and p-Akt inhibition by dasatinib (r = 0.843, p = 0.04). The inhibition levels of p-Src, p-Akt and p-FAK were measured when cells were treated with dasatinib at the dosage of 1uM and analyzed by ImageJ software.

The effects of dasatinib on Src and downstream targets were detected by western blotting in dasatinib-treated cells (Figure 4). The expression ratio of individual phosphor-protein to β-actin was quantified by ImageJ software (See Additional file 1). We analyzed the protein inhibition level in HCC cells when treated with dasatinib at the dosage of 1uM. In general, there was a significant correlation between the IC_50_ of dasatinib and the inhibition of p-Src (7/9, p < 0.05, Figure 5A), p-Akt (7/9, p < 0.05, Figure 5B) and p-FAK576/577 (7/9, p < 0.01, Figure 5C) by dasatinib. In all 3 sensitive cell lines, sk-hep1, Li-7 and PLC/PRF/6, the sensitivity to dasatinib was significantly correlated with p-Src and P-FAK576/577 inhibition by dasatinib. 5 out of 9 HCC cell lines including all sensitive cell lines had a significant correlation between p-Src inhibition and p-FAK576/577 inhibition by dasatinib (p < 0.05, Figure 6A). P-Src inhibition and p-Akt inhibition by dasatinib were also showed significant correlation in 5 HCC cell lines (p < 0.05, Figure 6B). We didn’t find any significant inhibition of Stat3 and MAPK42/44 activities in all cell lines by dasatinib at the dosage of 1uM and below (Figure 4).

**Figure 7:**
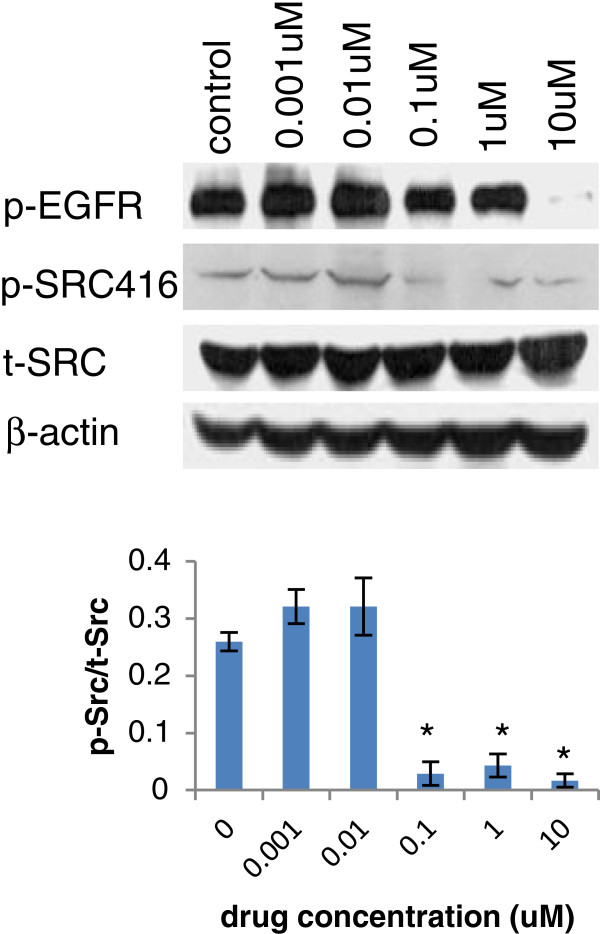
**Effect of dasatinib on PLC/PRF/6 cell signaling.** Total Src and p-Src, p-EGFR expression after dasatinib treatment in PLC/PRF/6 cell line were shown. P-Src/t-Src was quantified by ImageJ software. * *p < 0.05* as compared with the control (student’s *t*-test).

Individually, sk-Hep1, the most sensitive to dasatinib growth inhibition, showed only moderate inhibition of p- Src, p-FAK576/577 and p-Akt by dasatinib at the dosage of 1uM. . Even though dasatinib completely inhibited the expression of p-Src at 0.1uM in Li-7 cells, it only moderately reduced the p-FAK576/577 activity without inhibiting p-Akt (Figure 4); both sk-Hep1 and Li-7 expressed lower p-Src and p-Src/t-Src. It suggested that dasatinib may affect other signal pathway and inhibiting other protein kinase or growth factors to regulate cell growth in these two cell lines. PLC/PRF/6 was the only dasatinib sensitive cell line that co-overexpressed t-Src and t-EGFR, higher baseline expression of p-Src and lower p-Src/t-Src. In order to investigate whether dasatinib would affect EGFR signaling pathway, the activity of EGFR was tested too. The p-Src, p-FAK576/577, p-FAK861 and p-Akt were significantly inhibited by dasatinib at 0.1uM, p-EGFR1068 was inhibited at 10uM. No inhibition of t-Src expression by dasatinib at all (Figures 4 and 7). It appeared at lower concentration of dasatinib (0.01uM) there was a slight increase of p-Src. The mechanism of such difference is unknown. However, the ratio of p-Src/t-Src of control vs dasatinib treatment (0.01um) did not have any significant difference (p > 0.05).

Huh-7 was the least sensitive to dasatinib and very little level of p-Src was detected before dasatinib treatment but inhibition of p-Src can be demonstrated by dasatinib. In this cell line, dasatinib not only could not reduce p-FAK at both 576/577 and 861 sites, but also increased the level of them (Figure 4) suggesting Src dependant signaling pathway is not crucial in the regulation of oncogenic processes for Huh-7 cells.

HT-17 is one of the most resistant cell lines to dasatinib, but is sensitive to gefitinib [22]. It showed highest activity of EGFR at baseline. Even though dasatinib was able to inhibit p-Src416 at the lower dosage (1uM), but did not reduce p-Akt473 and P-MAPK42/44. These results indicated that the cell growth of HT-17 was most likely dependant on EGFR signal pathway.

**Figure 8:**
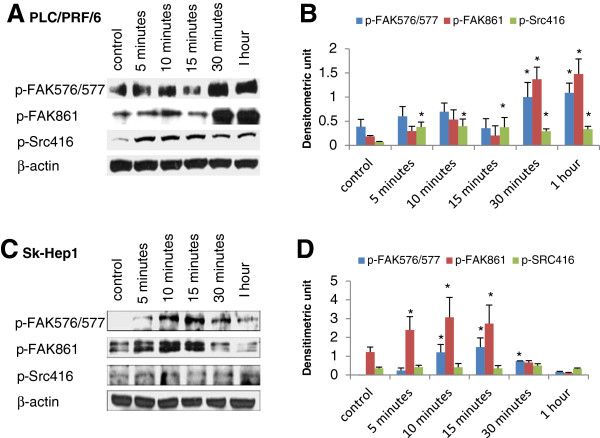
**The effect of EGF stimulation on phosphorylated protein expression.** PLC/PRF/6 cells were stimulated with 10 ng/ml EGF for the indicated times, lysed and analyzed by western blotting (**A**). Sk-Hep1 cells were stimulated with 200 ng/ml EGF for the indicated times, lysed and analyzed by western bloting with the indicated antibodies (**C**). The expression ratio of phosphorylated protein to β-actin was quantified by ImageJ software respectively. Results represented the mean (±SD) of three experiments. * *p < 0.05* as compared with the control (student’s *t*-test) (**B** and **D**).

Figure 8 showed that the response of phosphorylated proteins to EGF stimulation varied in different cell lines. P-Src can be activated by EGF (10 ng/ml) in PLC/PRF/6 (Figure 8A) but not in sk-Hep1 (Figure 8B). p-FAK 576/577, 861 can be activated by EGF in both cell lines. It suggested that FAK may be activated by other molecules such as the subunit PI3K p85, phospholipase Cr and Grb7 in sk-Hep1 cells [11].

### Dasatinib affects adhesion, migration and invasion of HCC cells

**Figure 9:**
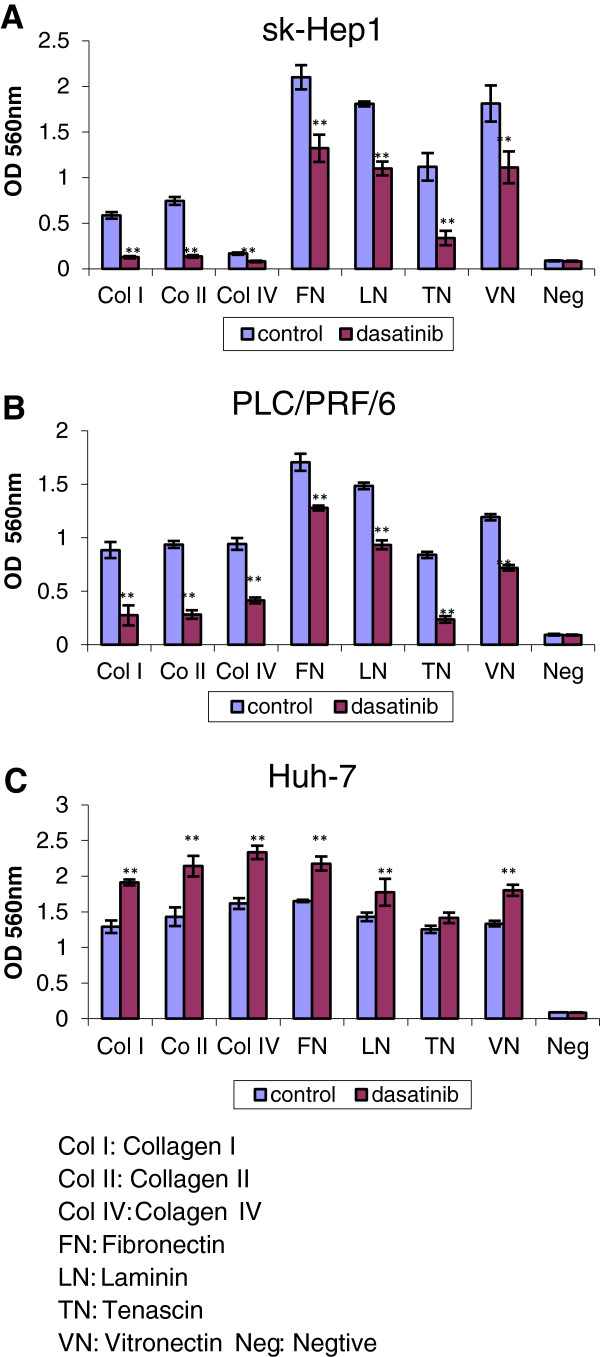
**The effect of dasatinib on cell adhesion in HCC cell lines (A**, **B**, **C).** Pretreatment for 24 hours, dasatinib inhibited adhesion of sk-Hep1 and PLC/PRF/6 cells on ECM protein (**A**, **B**), but increased the adhesion of Huh-7 cells (**C**). ** *p < 0.01* as compared with the control (student’s *t*-test).

There was a strong correlation between the p-FAK inhibition and cell adhesion, migration and invasion. After 24 h pretreatment, dasatinib significantly reduced adhesion of both sk-Hep1(p < 0.01) and PLC/PRF/6 (p < 0.001) on various ECM proteins (collagen I, collagen II, collagen IV, fibronectin, laminin, tenascin, vitronectin) with the range of inhibition from 25% to 82%, and the reduction percentages by dasatinib showed a similar pattern on both cell lines. However, in the most resistant cell line, Huh-7, the adhesion was significantly increased from 13% to 50% by dasatinib at the dose of 1uM (Figure 9, p < 0.01).

**Figure 10:**
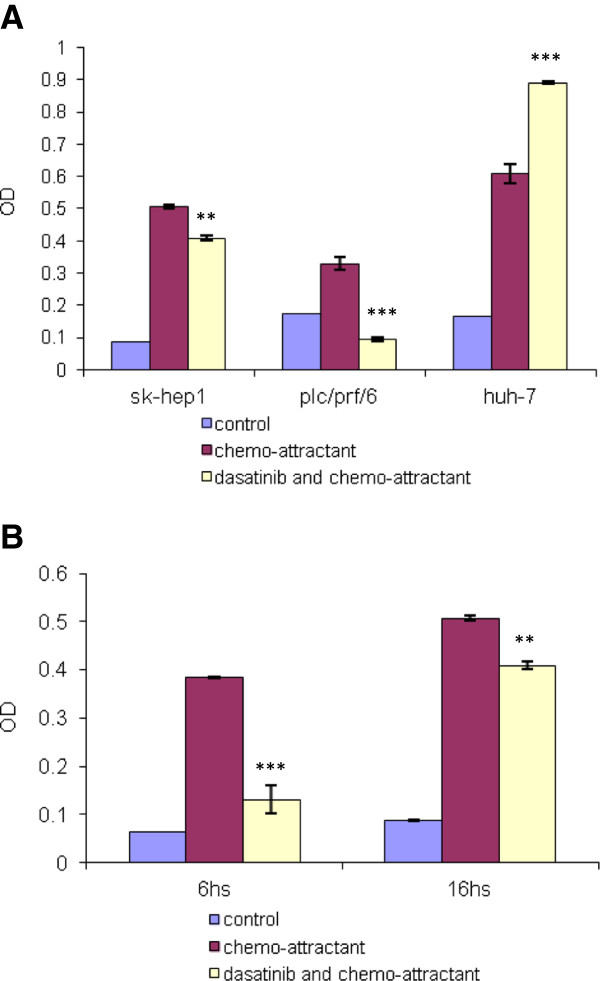
**The effect of dasatinib on cell migration in HCC cell lines. A**, dasatinib pre-treatment for 24 hours inhibited migration of sk-Hep1, PLC/PRF/6, but increased migration of Huh-7 cells. **B**, same test method as **A**, dasatinib inhibition on sk-hep1 cells 6 h and 16 h after removing dasatinib from media. The inhibitory effect was stronger at 6 h than that at 16 h. ** p < 0.01 and *** p < 0.001 as compared with the control (student’s *t*-test).

Dasatinib significantly reduced sk-Hep1 cells migration 6 h after removal from media (70% reduction as compared to control) (p < 0.001) but the inhibition of migration at 16 h was only 20% (Figure 10B). However, it reduced PLC/PRF/6 migration by 71% significantly at 16 h (p < 0.001). Again, Huh-7 cells migration was increased 50% by dasatinib (p < 0.001) (Figure 10A).

**Figure 11:**
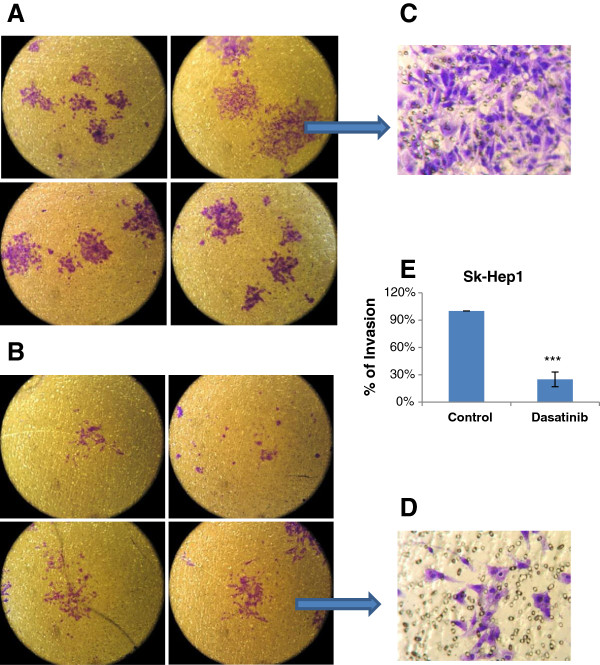
**Effect of dasatinib on cell invasion.** Invasive Sk-Hep1 HCC cells were captured in the polycarbonate membrane without (**A**) and with (**B**) the treatment of dasatinib at 1uM. Images were taken under invert light microscope (magnification: 100×). Images (**C**) and (**D**) were captured under high-power microscope (magnification: 600×). The cell numbers of at least 6 fields in each image were counted under microscope (magnification: 200×). The percentages of cell invasion were calculated (**E**). 3 independent experiments were carried out in duplicate. *** *p < 0.001* as compared with the control (student’s *t*-test).

Dasatinib significantly inhibited the invasion on ECM in sk-Hep1 cells (Figure 11, p < 0.001). Our results did not show any invasion inhibition by dasatinib in PLC/PRF/6 and Huh-7, however, PLC/PRF/6 and huh-7 were not invasive even in the absence of dasatinib.

## Discussion

In this report, we first demonstrated the heterogeneous sensitivity of 9 HCC cell lines to dasatinib in vitro as shown by their IC_50_ values. Our study also showed that the growth inhibition by dasatinib was correlated with t-Src in 7/9 cell lines and the p-Src/t-Src ratios were significantly lower in sensitive cells than resistant cells in the same 7/9 cell lines. In 6 resistant cell lines the growth inhibition by dasatinib was related to specific activity of Src protein by p-Src/t-Src ratio. With the exception of PLC/PRF/6, there was an inverse correlation between t-Src and t-EGFR. Song et al. showed that dasatinib treatment resulted in apoptosis in gefitinib-sensitive EGFR mutant lung cancer cells in-vitro [21]. Their findings were also confirmed by other investigators recently [23,24]. Our results showed even in gefitinib resistant HCC cell lines [22], some were still sensitive to dasatinib. There was also a co-overexpression with Src and members of EGFR family in breast cancer [25]. Our findings that EGFR expression influenced the response of HCC cells to dasatinib further strengthened the notion that a unique cross-talk mechanism might exist between Src family and EGFR family tyrosine kinases in hepatocarcinogenesis. These two TK signaling pathways may complement each other in the oncogenic process and development of resistance to treatment of either pathway. Our results suggested combination of inhibitors of both pathways may yield better results, as we have shown synergistic interaction between dasatinib and gefitinib in HCC cells on our previous study [22]. The preliminary study of dasatinib and erlotinib (an EGFR TKI) combination in 29 evaluable patients with recurrent or metastatic non-small cell lung cancer showed 2 partial response and 62% disease control rate [26]. More studies are needed to explore the optimal combination and the right clinical settings.

Baseline t-Src and specific Src activity (p-Src/t-Src) may be used as useful predictive biomarkers for selecting dasatinib treatment in HCC patients. We also showed in most of cell lines, dasatinib suppressed the expression of p-Src, p-FAK and p-Akt which correlated with the level of growth inhibition. So the inhibitory response of p-Src, p-FAK and p-Akt to dasatinib may also provide guidance for predicting response, although they were more variable than baseline t-Src. Significant correlation between IC_50_ and expression of t-Src could be shown in majorities of cell lines, especially in gefitinib resistant cell lines. However, there were exceptions, such as Huh-7 cells, Src-dependant signal pathway was not an important determinant of cell proliferation, motility and invasion in Huh-7 cells which was resistant to dasatinib but showed p-Src inhibition by dasatinib. Interestingly, we found that high ratio of p-Src/t-Src was significantly associated with less resistant to dasatinib in all 6 dasatinib resistant cell lines. This implied that the mechanism of action of dasatinib in sensitive cell lines may be different from that of resistant cell lines. In addition, there were differences among other cell lines in the inhibition of p-Src, p-FAK, p-Akt, cell adhesion, migration and invasion by dasatinib. Thus, we demonstrated the heterogeneity of HCC tumor biology and the need for individualized treatment. Biomarkers may provide guidance for selecting right treatment for the right patient. It will require prospective studies to validate our findings. In the study of combination of dasatinib and erlotinib in patients with advanced NSCLC, reduction of vascular endothelial growth factor (VEGF) was correlated with disease control [26]. However, a phase II study of single agent dasatinib in advanced NSCLC showed that neither activation of SFK nor EGFR and Kras mutations in tumor tissue predicted response to dasatinib [27]. No clinical results are available yet from studying dasatinib in advanced HCC patients.

Src interacts with FAK to play a key role in tumor cell migration and invasion. Upon intergrin engagement or stimulation of EGF or PDGF receptors, FAK autophosphorylates at pTyr397, creating a high affinity binding site for Src, the association between Src and FAK resulted in activation of Src and phosphorylation of FAK at Tyr 576, 577, 861 and 925. The Src/FAK complex phosphorylated a number of other focal adhesion proteins and activated other intra cellular signaling pathway [28]. This interaction between Src and FAK has been shown to control both cell motility and invasion [11]. Regarding our results, in 56% (5/9) studied HCC cell lines, dasatinib inhibits the activity of Src to reduce phosphorylation of FAK. Inhibition of FAK at Tyr576/577 was strongly correlated with HCC cell adhesion, migration and invasion. For 78% (7/9) of studied HCC cell lines, reduction of activated FAK576/577 was significantly correlated with the dasatinib sensitivity. Thus the SFK/FAK signaling pathway plays an important role in cell adhesion, migration and invasion. Inhibition of this pathway is one of the mechanisms of action of dasatinib. In MDA-MB-231 human metastatic breast cells, dasatinib also showed the inhibition of cell proliferation, migration and invasion, as well as the inhibition of Src, Fak (Y925), paxillin, caveolin-1 and p130Cas activation [29]. Furthermore, conditional expression of SrcDN in MCF7 human breast cancer cells reduces adhesion, migration and spreading. Because expression of SrcDN alters the shape of MCF7 cells, immunofluorescence confocal analyses showed concentrated focal adhesion proteins. However, the adhesion of cells was reduced [30]. In contrast, the most resistant HCC cell line Huh-7 expresses escalated levels of activated FAK576/577 and increases cell adhesion and migration after dasatinib treatment. A previous study reported that increased cell adhesion, migration occured at the same time upon treatment with prostaglandin E2by mediating FAK/paxillin/Erk2 signal pathway in the same HCC cell line (Huh-7) [31]. The mechanism of dasatinib induced increases of cell adhesion, migration in Huh-7 cells need further investigation. However, the nature of cell origin may determine specific cellular responses and the activated FAK576/577 may be the factor contributing to drug resistance.

Our study also revealed that FAK can be activated by EGF in HCC cell lines. In PLC/PRF/6 cell line, Src and FAK can be activated simultaneously by EGF, and completely inhibited by dasatinib. In view of this result, dasatinib may directly inhibit the complete activation of FAK through reducing the activity of Src TK. For sk-Hep1 cell line, EGF could not activate Src, but dasatinib could also reduce the activity of FAK, indicating dasatinib may interplay with other molecules to block the phosphorylation of FAK, and therefore inhibit the motility and invasion of HCC cells.

The activated PI3K/PTEN/Akt/mTOR pathway has emerged as a novel contributor to HCC tumor development [12]. 56% (5/9) of our studied HCC cell lines showed the inhibition of Src activity by dasatinib also induced inhibition of p-Akt. It suggested that activated Src might trigger PI3K pathway to activate Akt, which regulated multiple cellular proteins in cell proliferation, apoptosis, metastasis and angiogenesis. In PLC/PRF/6 cell line, complete inhibition of activated Src by dasatinib at the dosage of 0.1 uM, not only induced the inhibition of Akt activity at the same dosage, but also induced the inhibition of p-EGFR at Tyr1068 at higher dosage of 10uM (Figure 6). These findings indicated that EGFR may be a direct target of dasatinib or an indirect target secondary to Src inhibition [8,11].

Our data showed little inhibition of p-Stat3, and p-MAKP 42/44 by dasatinib in all HCC cell lines except at high concentration. Activation of Stat3 by altered Janus-activated Kinase-Stat3 binding has been reported as a potential mechanism of resistance to Src inhibition [32] and should be a focus of future research on mechanisms of dasatinib resistance. In the resistant Huh-7 cells, p-Stat3 expression was not different from sensitive cell lines, suggesting Stat3 may not play an important role in this cell line. Dasatinib was synergistic with oxaliplatin against colon carcinoma cells and with cisplatin against NSCLC cells [33,34]. It was also synergistic with gefitinib, bravinib, BMS-690514, BMS-536924 or ixabepilone as shown in our previous studies [22]. In the future, it may be necessary to perform genomic and proteomic evaluation of each patient to determine resistance patterns as shown by Li et al. that dasatinib had nearly 40 distinct kinase targets [35,36].

## Conclusions

Dasatinib inhibits the proliferation, adhesion, migration and invasion of HCC cells in vitro via inhibiting Src and affecting SFK/FAK and PI3K/PTEN/Akt signaling pathways, but not Ras/Raf/MEK/ERK and JAK/Stats pathways. Apart from Src, dasatinib may also inhibit other tyrosine kinase protein or growth factor receptors in HCC cells. In general the growth inhibition by dasatinib was related t-Src and the ratio of p-Src/t-Src. T-Src and p-Src/t-Src may be useful biomarkers to select HCC patients for dasatinib treatment in the future. This is consistent with the notion that the Src family Kinases cooperate with multiple receptor tyrosine Kinases to modulate signaling cross talk and promoting proliferation, adhesion, migration and invasion. Furthermore, dasatinib could be an attractive agent for combination therapies such as combining with EGFR TKI or chemotherapy to exploit potential synergistic interaction. Hence, further laboratory and translational researches are warranted to investigate the role of dasatinib or other Src inhibitor in HCC.

## Competing interests

Alex Y. Chang serves as a member of advisory committees of: Eli Lilly, Astella Pharma Inc., Eisai Limited, Bristol Myers Squibb Company, Agennix Inc. and received Research funding from Astella Pharma Inc., Eisai Limited, Bristol Myers Squibb Company, Roche, Agennix Inc. for conducting clinical trials.

## Authors’ contributions

Study conception and design: AYC Performing tests: WM Analysis and interpretation: AYC, WM Drafting manuscript: AYC, WM. Both authors read and approved the final manuscript.

## Pre-publication history

The pre-publication history for this paper can be accessed here:

http://www.biomedcentral.com/1471-2407/13/267/prepub

## Supplementary Material

Additional file 1 **Densitometric quantitation of the blots of Figure 4.** The expression ratio of phosphorylated protein to β-actin was quantified by ImageJ software respectively. Results represented the mean (±SD) of three experiments. * *p < 0.05* as compared with the control (student’s *t*-test). Click here for file
